# Development of a new affinity maturation protocol for the construction of an internalizing anti-nucleolin antibody library

**DOI:** 10.1038/s41598-024-61230-z

**Published:** 2024-05-08

**Authors:** Rita Ribeiro, João N. Moreira, João Goncalves

**Affiliations:** 1 CNC—Center for Neurosciences and Cell Biology, Center for Innovative Biomedicine and Biotechnology (CIBB), Faculty of Medicine (Polo 1), University of Coimbra, Coimbra, Portugal; 2 Faculty of Pharmacy, iMed.ULisboa - Research Institute for Medicines, University of Lisbon, Lisbon, Portugal; 3 Univ Coimbra—University of Coimbra, CIBB, Faculty of Pharmacy, Coimbra, Portugal

**Keywords:** Drug development, Antibody therapy, Protein design

## Abstract

Over the last decades, monoclonal antibodies have substantially improved the treatment of several conditions. The continuous search for novel therapeutic targets and improvements in antibody’s structure, demands for a constant optimization of their development. In this regard, modulation of an antibody’s affinity to its target has been largely explored and culminated in the discovery and optimization of a variety of molecules. It involves the creation of antibody libraries and selection against the target of interest. In this work, we aimed at developing a novel protocol to be used for the affinity maturation of an antibody previously developed by our group. An antibody library was constructed using an in vivo random mutagenesis approach that, to our knowledge, has not been used before for antibody development. Then, a cell-based phage display selection protocol was designed to allow the fast and simple screening of antibody clones capable of being internalized by target cells. Next generation sequencing coupled with computer analysis provided an extensive characterization of the created library and post-selection pool, that can be used as a guide for future antibody development. With a single selection step, an enrichment in the mutated antibody library, given by a decrease in almost 50% in sequence diversity, was achieved, and structural information useful in the study of the antibody-target interaction in the future was obtained.

## Introduction

Monoclonal antibodies represent a relevant fraction of the therapeutic arsenal available nowadays, particularly in the treatment of infectious diseases, immune-mediated disorders, and cancer^1^. Current research is focused on the development of antibodies against novel targets, along on the modulation of affinity and specificity, while ensuring the best efficacy and safety profiles^2^.

Affinity is associated with the antibody-antigen binding strength, which is directly correlated with the therapeutic potency of the molecule^3^. Amongst other factors, such as antigen density and binding kinetics, it depends greatly on the structures of the molecules involved in the binding interaction^4^. For antibodies capable of Fc-mediated immune functions, such as Antibody-Dependent Cell-mediated Cytotoxicity (ADCC), modulation of the affinity to the target antigen is an attractive approach, as it has been demonstrated that moderate to high values of affinity are required for effective activity^2,5–8^.

In this regard, affinity maturation is a process that in vivo takes place through hypermutation and clonal selection in secondary lymphoid organs, and has been successfully recapitulated in vitro to increase affinity in antibody development^9–13^. In vitro affinity maturation comprehends the construction of antibody libraries, followed by screening and selection of the clones of interest. Modern approaches, such as Next Generation Sequencing (NGS) and computational tools, enable a deeper characterization of the libraries and a rational design of the molecules, complementing antibody development^14^.

Nowadays, large antibody displaying libraries (> 10^11^ elements) can be constructed recurring to synthetic methods, such as site-directed or random mutagenesis^15^. While the first requires an extensive knowledge about the target’s structure in order to introduce rationally designed mutations which contribute to specified properties (for example, binding specificity), the latter promotes unspecified nucleotide misincorporation in the antibody coding DNA^16^. Despite the risk that randomly introduced mutations may impair antibodies' characteristics, such as solubility or immunogenic potential, they allow a wide spectrum of mutations along the antibody molecule, culminating in the construction of much diverse libraries^14^. Random mutagenesis comprises in vitro or in vivo approaches, including error-prone Polymerase Chain Reaction (PCR), degenerate oligonucleotides-Pfu (DOP), or mutator bacterial strains. Although the use of bacterial strains promotes lower mutation frequencies, it is the simplest method to perform, as it eliminates the need for primers design, and the subcloning step necessary to proceed with the selection process^17^. *Escherichia coli* mutator strain XL1-Red has been commonly used in library construction, but it presents low mutational potency and poor transformation efficiency^18^. Other *E. coli* strains have been developed to enhance mutational frequency, but these options increased mutagenesis spectrum along the target genes^19,20^. In order to address some of these challenges, herein, an *E. coli* JS200 strain will be used to create diversity in an anti-nucleolin antibody previously developed by our group^21^. It harbors a low-fidelity DNA polymerase I that has a mutation rate comparable to the XL1-Red strain^22,23^, but allows a mutational hotspot close to the ColE1 origin of replication, thus introducing mutations preferentially in our antibody coding fragment^24^.

The second phase of affinity maturation consists in the selection of clones of interest by antibody display and screening. Phage display, firstly introduced by George Smith in 1985^25^, remains the most common selection technique due to its robustness, simplicity, and inexpensiveness. Its success is translated in blockbuster drugs, such as adalimumab and atezolizumab^26^. The convenience of this technique relies on the strong correlation between genotype and phenotype in bacteriophage assembly, enabling an easy expression of antibody libraries in fusion to phage coating proteins^27^. It is particularly useful in the development of small antibody fragments^28^, such as the VHH (single variable domain of camelid heavy chain immunoglobulins) used herein, as they can be easily and stably displayed at phage surface, along with the low propensity to aggregate^29,30^. Moreover, antibody fragments can be transformed into full IgG or VHH-Fc formats by recombinant technology, acquiring additional functionalities, without losing specificity^21,31–33^.

A successful phage display selection demands an adequate selective pressure and biopanning optimization^28^. The biopanning method requires that the antigen structure remains as close to its native conformation as possible, so that the antibody-antigen interaction mimics the in vivo setting. The antibody target used in this work is nucleolin, a protein present at the cell surface of highly proliferating cellular components of the tumor microenvironment^34,35^, that acts as a co-receptor in the binding and/or internalization of a range of molecules involved in angiogenesis and tumor growth^36–38^. Nucleolin’s full length tertiary structure is yet to be fully characterized^39^, which hinders the use of the isolated protein for panning. In this regard, whole cell-based phage display has the advantage of presenting proteins in their native conformation and level of expression at the cell surface^40^, takes into account interaction with neighboring proteins, while adding the possibility of selecting both internalizing^41–44^ or non-internalizing agents^45–48^. Screening by phage internalization is an attractive strategy to select antibodies against intracellular targets, or antibodies that act as intracellular carriers, such as antibody–drug conjugates. In line with this, NGS technologies have been adapted for the in-depth evaluation of the antibodies complementarity determining region (CDR) sequences^49^. Data collected from NGS has served for library characterization^50–53^, and as a guideline for further selection^49,54^.

An anti-nucleolin VHH was previously developed by our group^21^, by engrafting the CDR3 region of a VHH scaffold with a ten amino sequence from the F3 peptide, known to bind cell surface nucleolin^55^. Using this antibody as reference, herein a fast and simple method of antibody library construction and phage screening to be used in affinity maturation protocols is proposed. The use of a mutator bacteria strain not described before for antibody library construction, coupled to a cell-based phage display library screening adapted to the selection of cancer cells-internalizing antibody clones, will be tested for the first time. It is hypothesized that such procedure will enable the generation of an antibody pool that can be internalized by tumor nucleolin-overexpressing cells.

## Results

### Construction and characterization of anti-nucleolin VHH antibody library by in vivo random mutagenesis

In vivo random mutagenesis was performed on a reference anti-nucleolin VHH antibody, based on a construct previously developed by our group^21^. Reference anti-nucleolin VHH coding fragment was cloned in a pComb3X vector to facilitate further construction of a phage library. The resulting reference plasmid was transformed into JS200 bacteria, a mutator strain of *E. coli* that harbors a low-fidelity DNA polymerase I. At 37 °C and under restrictive conditions (saturated culture), this enzyme promotes random mutagenesis in ColE1 plasmids, such as pComb3X. After four rounds of iterative random mutagenesis, an antibody library of mutated VHHs was constructed. This library has a theoretical size of 2.19 × 10^8^ transformants, a value extrapolated from the transformation efficiency of ER2738 bacteria with DNA collected after the 4th round of mutagenesis. However, this traditional method of evaluating library diversity does not provide information on the quality and functional complexity of the library. Therefore, Next Generation Sequencing was performed to characterize the VHH library regarding its diversity (Table 1), as well as to validate the mutagenesis method used.

**Table 1:** Characterization and comparison of pre- and post-selection libraries.

	Pre-selection library (%)	Post-selection library (%)
Raw reads	14,025	39,890
Merged reads	9683	31,322
Nucleotide clusters	8373 (86.47% of merged)	18,454 (46.26% of merged)
Functional sequences^a^	7581 (78.29% of merged)	23,317 (74.44% of merged)
*Sequences with low quality*^b^	2102 (21.71% of merged)	8005 (25.56% of merged)
Amino acid clusters	5249 (69.24% of translated)	8346 (35.79% of translated)
*Single occurrence amino acid sequences*	4842 (92.25% of clusters; 63.87% of translated)	7246 (86.82% of clusters; 31.08% of translated)
*Clusters with repeated amino acid sequences*	407 (7.75% of clusters, 5.37% of translated)	1100 (13.18% of clusters; 4.72% of translated)
DNA diversity^c^	86%	59%
Protein diversity^d^	69%	36%

^a^Translated sequences without stop codons and determined in the correct frame.

^b^Difference between merged and functional sequences.

^c^Percentage of nucleotide clusters in the library, calculated using the ratio between nucleotide clusters (i.e. different sequences in the pool) and merged reads (total sequences).

^d^Percentage of amino acid clusters in the library, calculated using the ratio between amino acid clusters (i.e. different sequences in the pool) and merged reads (total sequences).

About 1.5 µg of DNA corresponding to the VHH fragment of the library were sequenced giving a total of 14,025 reads. Raw reads were quality-filtered to remove sequencing adapters, reads with less than 150 bases, as well as bases with an average quality lower than Q25. Then, the forward and reverse reads were merged, resulting in 9683 VHH sequences (Table 1). For further calculations, the number of merged sequences was considered as the total number of sequences in the analyzed pool, since the raw sequences included fragments that did not correspond to entire sequences, and to sequences with low quality.

The merged sequences were translated into amino acid sequences, for further analysis of the functional complexity of the antibody library. Sequences with stop codons, that would impair the production of functional and soluble antibodies, were filtered out, resulting in 7581 protein sequences (78.29% of the merged sequences; Table 1). This low frequency of stop codons and frameshift mutations, and the similarity between the consensus sequences of the “translated” and the “translated without stop codons”, evidenced that there were no significant frameshift mutations with an impact in the translation of most sequences, and were indicative of the good quality of the library. Additionally, only 4% of the VHH sequences presented a size different from the reference sequence, suggesting that most mutations were base transitions, instead of insertion or deletions.

Within these high-quality sequences, 5249 clusters were identified, demonstrating that 69.24% of translated sequences were unique, and that the antibody library incorporated a functional diversity of 69%. Clusters consisting in single copy sequences were estimated to be 92.24%, while the remaining 7.76% corresponded to clusters with more than one copy of the same sequence (Table 1).

MiSeq Illumina technology can cover the whole extent of the VHH’s 371 bp, as it reads up to 600 bp in a single read^54^. It was thus possible to sequence the whole VHH fragment, and to analyze the mutated antibody library. Regions of the VHH with the highest Percentage of Identity (PID) corresponded to CDR1 and CDR2 regions, with more than 95% PID for each individual nucleotide. Lowest consensus corresponded to the CDR3 region. Additionally, as the CDR3 is the antibody region with higher variability and most involved in binding to the antigen^56^, and the one whose diversity has been proven to be determinant in antibody library selection^57^, an analysis of its diversity was performed.

**Figure 1:**
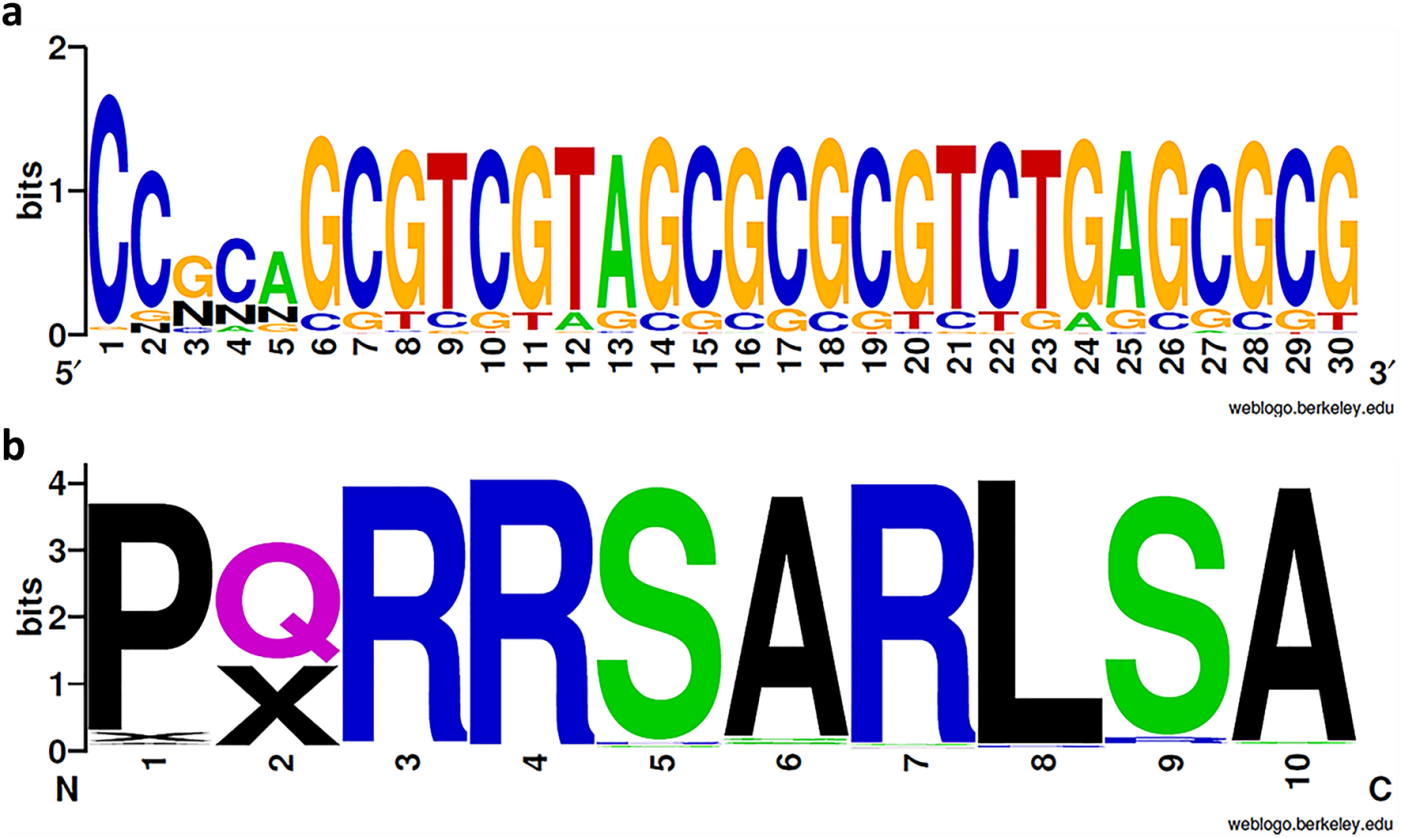
WebLogo representations of the merged CDR3 region nucleotide sequence (**a**) and amino acid sequence (**b**) from the pre-selection library. The height of each stack represents the conservation at that position, measured in bits, and the height of each symbol is proportional to its frequency at that particular position. C—Cysteine, G—Guanine, A—Adenine, T—Thymine, N—Unidentified nucleotide, P—Proline, Q—Glutamine, R—Arginine, S—Serine, A—Alanine, L—Leucine, X—Unidentified amino acid.

In the DNA library, 3103 reads were equal to the reference CDR3, corresponding to 32.06% of the merged reads. Sequences coding for the CDR3 region had an average consensus of 83.27%, with a PID between 55.26 and 87.4% for each nucleotide of the region. Nucleotides in positions 3, 4 and 5 of the VHH fragment incorporated the highest variability, with PID of 55.26%, 67.34% and 63.53%, respectively. Despite a considerable percentage of unidentified nucleotides (N) (33.11%, 20.98%, and 21% for positions 3, 4, and 5, respectively), the most frequent mutations determined for these positions were transition from guanidine (G) to cytosine (C) in position 3 (8.95%), from C to adenine (A) in position 4 (8.34%), and from A to G in position 5 (12.04%) (Fig. 1a).

Regarding the peptide library, the reference CDR3 appeared in 2447 clusters, representing 46.62% of the functional sequences. The CDR3 region presented the highest diversity in the second amino acid (Fig. 1b), in accordance with the nucleotide analysis. Following the aforementioned frequency of unidentified nucleotides (N) at positions 3, 4 and 5 of the CDR3 DNA sequence, a frequency of 38.67% of unidentified amino acids (X) was observed in position 2. As such, it was not possible to predict the amino acids that were replacing the original glutamine in the second position of the CDR3 amino acid sequence. The five most frequent mutations in the library were serine to arginine in position 9 (3.26% of clusters), alanine to glycine (1.87%) or serine (1.75%) in position 6, serine to arginine in position 5 (1.58%), and proline to arginine in position 1 (1.39%).

### Construction of phage library and labeling with pH sensitive dye

Library’s good quality was an important prerequisite for further transformation into a phage library, since sequences with stop codons or frameshift mutations may result in non-functional antibodies, sequences with incorrect folding, or clones with solvent-exposed hydrophobic amino acids with propensity for aggregation. This can result in inefficient display of the antibodies at the phage surface and, consequently, prevalence of phages displaying undesirable antibody variants, which compete with the positive clones, affecting the size and functionality of the phage library^15^. Therefore, a phage library displaying the mutated antibody clones was created, by performing four parallel transformation reactions of ER2738 bacteria with the DNA obtained after the 4th round of mutagenesis. Phages displaying the reference anti-nucleolin VHH were also produced to serve as control in further experiments.

**Figure 2:**
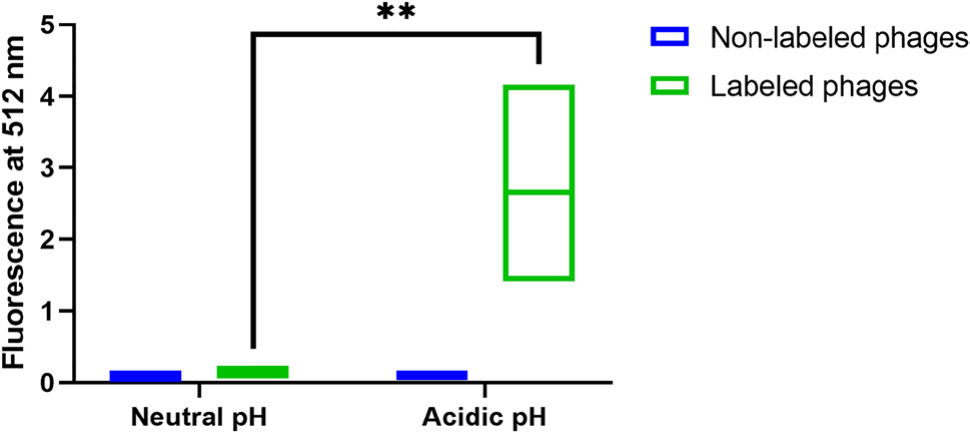
Fluorescence signal of phages labelled with pH-sensitive dye pHrodo as function of pH. Phages displaying the reference antibody were labeled with pH sensitive dye pHrodo Green and used as coating in ELISA plates. After being incubated with neutral (pH 7.4) or acidic (pH 4) solutions and washed twice with PBS, fluorescence was measured at 512 nm. A control experiment using non-labeled phages was performed in parallel. Data represent the mean of three independent experiments, performed in duplicate. Differences in emitted fluorescence were evaluated by two-way ANOVA followed by Tukey’s test (*p < 0.05, **p < 0.01, ***p < 0.001).

Phages displaying the mutated antibody library and phages displaying the reference VHH were then labeled with pH-sensitive pHrodo™ iFL Green STP ester, amine-reactive dye. Non-labeled and labeled phages were incubated in acidic or neutral solutions, to confirm whether phage labeling took place. As expected, fluorescence emitted by pHrodo labeled phages in an acidic environment was significantly higher than the one emitted when in neutral solution (Fig. 2). Non-labeled phages used as negative control did not show any signal upon exposure to either neutral or acidic conditions (Fig. 2). These results confirmed that the labeling reaction was effective and stable.

### Selection of cell internalizing library-displaying phages

**Figure 3:**
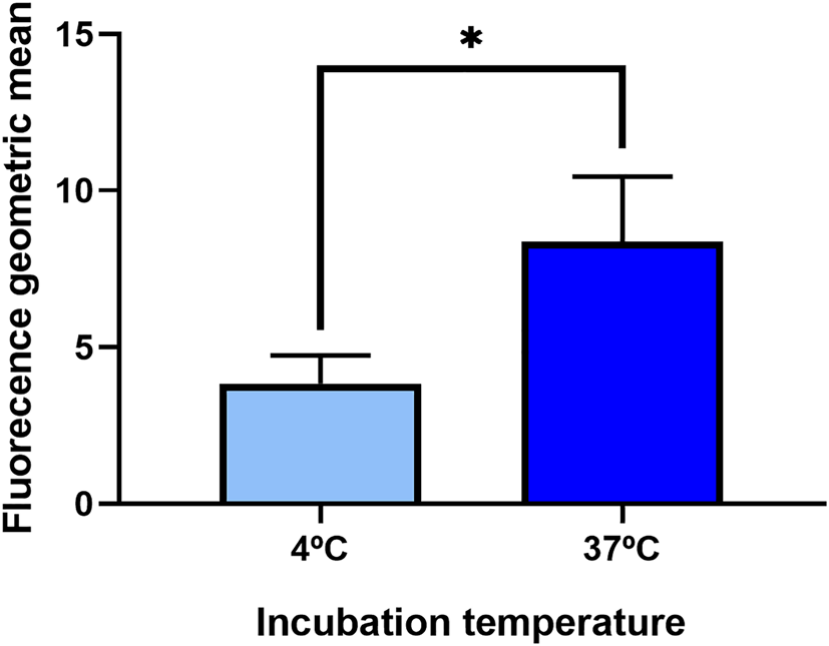
Interaction of nucleolin-overexpressing cells with pHrodo-labeled phages displaying the reference anti-nucleolin antibody under non-internalizing (4 °C) or internalizing (37 °C) conditions. Incubations at both temperatures were performed in parallel experiments, and further analyzed by flow cytometry Data represent the mean of three independent experiments. Differences in emitted fluorescence were evaluated by Unpaired t-test (*p < 0.05, **p < 0.01, ***p < 0.001).

Once the phage labeling was validated, preliminary flow cytometry experiments were performed to evaluate the feasibility of further cellular internalization-based selection by Fluorescence-Activated Cell Sorting (FACS). First, nucleolin-overexpressing cells were incubated with phages displaying the reference anti-nucleolin antibody under non-internalizing (4 °C) and internalizing conditions (37 °C) to assess nucleolin-mediated internalization, a feature described for other anti-nucleolin ligands^35,58^ and M13 bacteriophages^59^. The significant two-fold difference (*p* < 0.05) of the fluorescence signal between the two conditions tested supported both cellular internalization and exposure to an acidic environment of the phages labelled with the pH-sensitive probe (Fig. 3).

**Figure 4:**
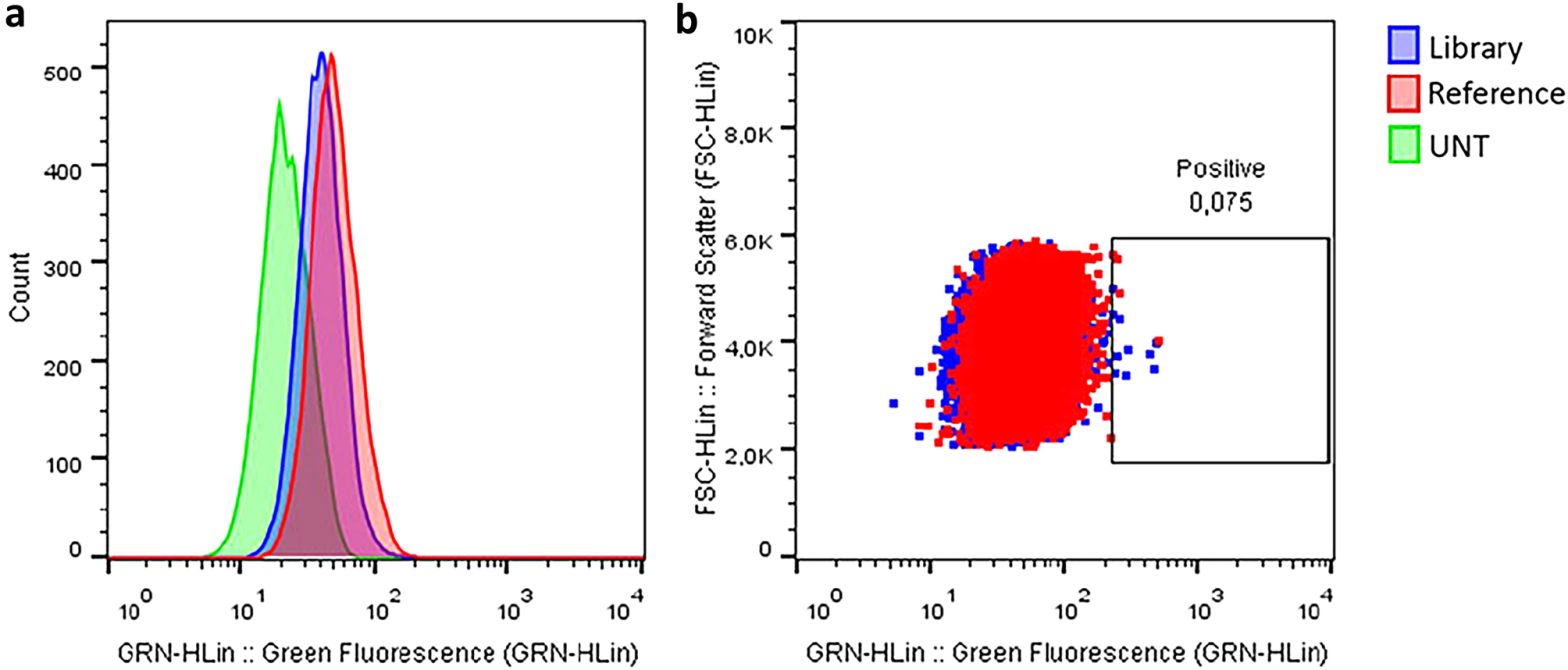
Interaction of pHrodo labeled phages displaying either the constructed antibody library (blue) or the reference antibody (red) with nucleolin-overexpressing cells, under internalizing conditions (37 °C). After analysis by flow cytometry, green fluorescence emitted by internalized labeled phages was represented in the form of histogram (**a**) and dot plot (**b**). Data result from a representative experiment. Green—Untreated cells.

We then hypothesized that events corresponding to higher fluorescence signal have internalized phages displaying antibody clones with higher affinity to nucleolin compared to phages displaying the reference antibody. Results from a representative experiment demonstrated that, despite a high extent of overlap between the two populations (Fig. 4a), it is possible to distinguish 0.075% of the collected events with increased fluorescence in the mutated library (Fig. 4b). Notwithstanding the reduced percentage, the fact that these events exhibited increased fluorescence in one order of magnitude superior to the main population, prompted us to proceed to the sorting experiment.

**Figure 5:**
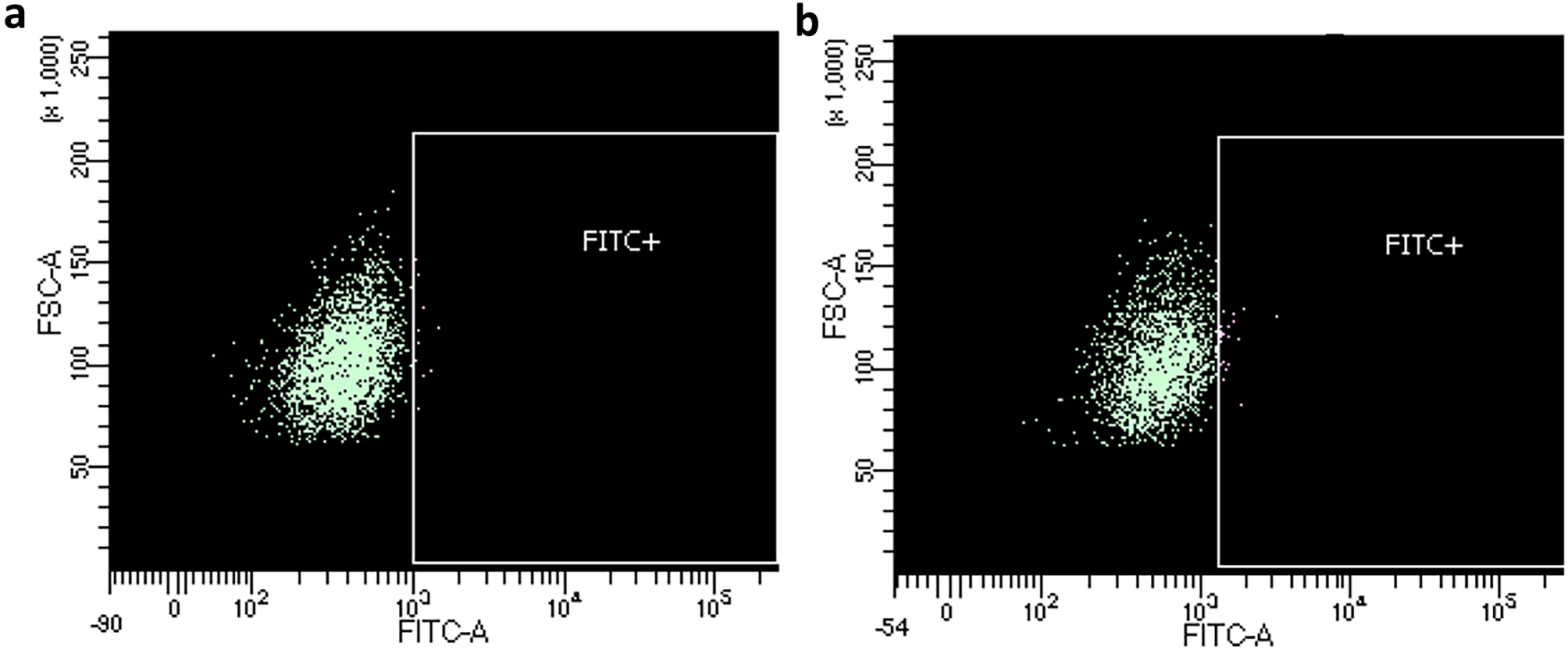
Representative scatter plots of either labeled reference antibody- (**a**) or library-displaying phages (**b**). Nucleolin-overexpressing cells were incubated with the referred labeled phages at 4 °C for 45 min, followed by two washing steps, and a final incubation at 37 °C for 1.5 h. In both scatter plots, the FITC + gate was considered positive for the highest extent of internalization, and the corresponding phages were collected for further analysis. Data represent one of two experiments performed in parallel.

After demonstration that labeled phages displaying both the reference antibody or the mutated library could be internalized by nucleolin-overexpressing cells, conditions were set to select events of interest by FACS. Two experiments were performed in parallel to increase the likelihood of sorting positive clones. After selection of the positive population by comparison against the control cells incubated with phages displaying the reference antibody (Fig. 5a), a total of 706 events were sorted in Phosphate-Buffered Saline (PBS) (Fig. 5b).

Library-displaying internalized phages were then obtained by cell lysis and used to infect ER2738 bacteria. Colonies were collected for phage plasmid extraction and the resulting DNA constituted the sorted mutated antibodies pool (henceforth called post-selection library).

### Characterization of the selected library

**Figure 6:**
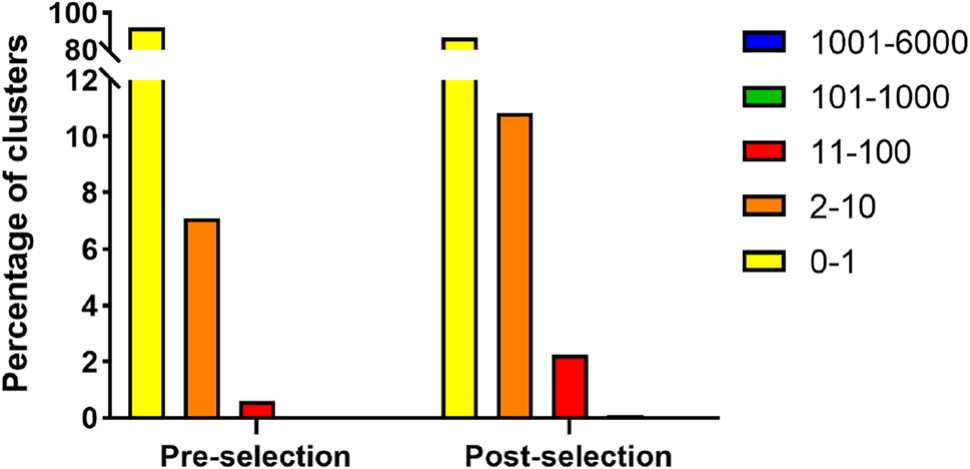
Comparison between the percentage of clusters with different number of sequence copies in the pre- and post-selection libraries. NGS analysis of VHH fragments from the original antibody library and the post-selection antibody pool included the clustering of amino acid sequences, thus allowing the identification of antibody clusters. Each cluster corresponds to a different amino acid sequence, and may be composed of a single or multiple copies of that same sequence.

Post-selection library corresponds to VHH DNA fragments amplified by PCR from phage DNA collected from sorted cells. About 6 µg of DNA were sequenced using NGS technology MiSeq Illumina, originating a total of 39,890 reads. After quality filtering and merging, 31,322 reads were obtained, and translated into amino acid sequences. Functional sequences, i.e., amino acid sequences without stop codons, comprised 74.44% of the reads, thus reinforcing the quality of protein pool. Several amino acid clusters were identified (8436), suggesting that 35.79% of the sorted pool corresponded to different amino acid sequences, against the 69.24% of the pre-selection library, indicating a half decrease of the diversity, approximately, following the selection. Amongst these, 7246 clusters were single occurrence sequences, representing 86.82% of the clusters, and 31.08% of the translated pool. Thus, although with a still relatively high percentage of single copy sequences in the total pool, these significantly decreased to half in the translated pool, showing that many of the mutated antibodies were eliminated upon selection. Clusters with more than one copy of the same sequence increased from 7.75 to 13.18%, suggesting an enrichment in certain sequences (Fig. 6). The five clusters with the highest number of copies in both pre- and post-selection libraries, all have a percentage of similarity higher than 96% with the reference sequence, and mutations were only identified in nucleotides outside the VHH sequence, in the PCR primers binding sites, suggesting that many other VHH fragment clusters may actually represent the reference sequence. Table 1 presents a compilation of the post-selection NGS data analysis, allowing a better comparison between pre- and post-selection libraries.

Similar to the pre-selection antibody library, we proceeded to analyze the CDR3 coding region of the post-selection pool, to understand which mutations were advantageous in nucleolin binding and whether there was an enrichment in these sequences.

**Figure 7:**
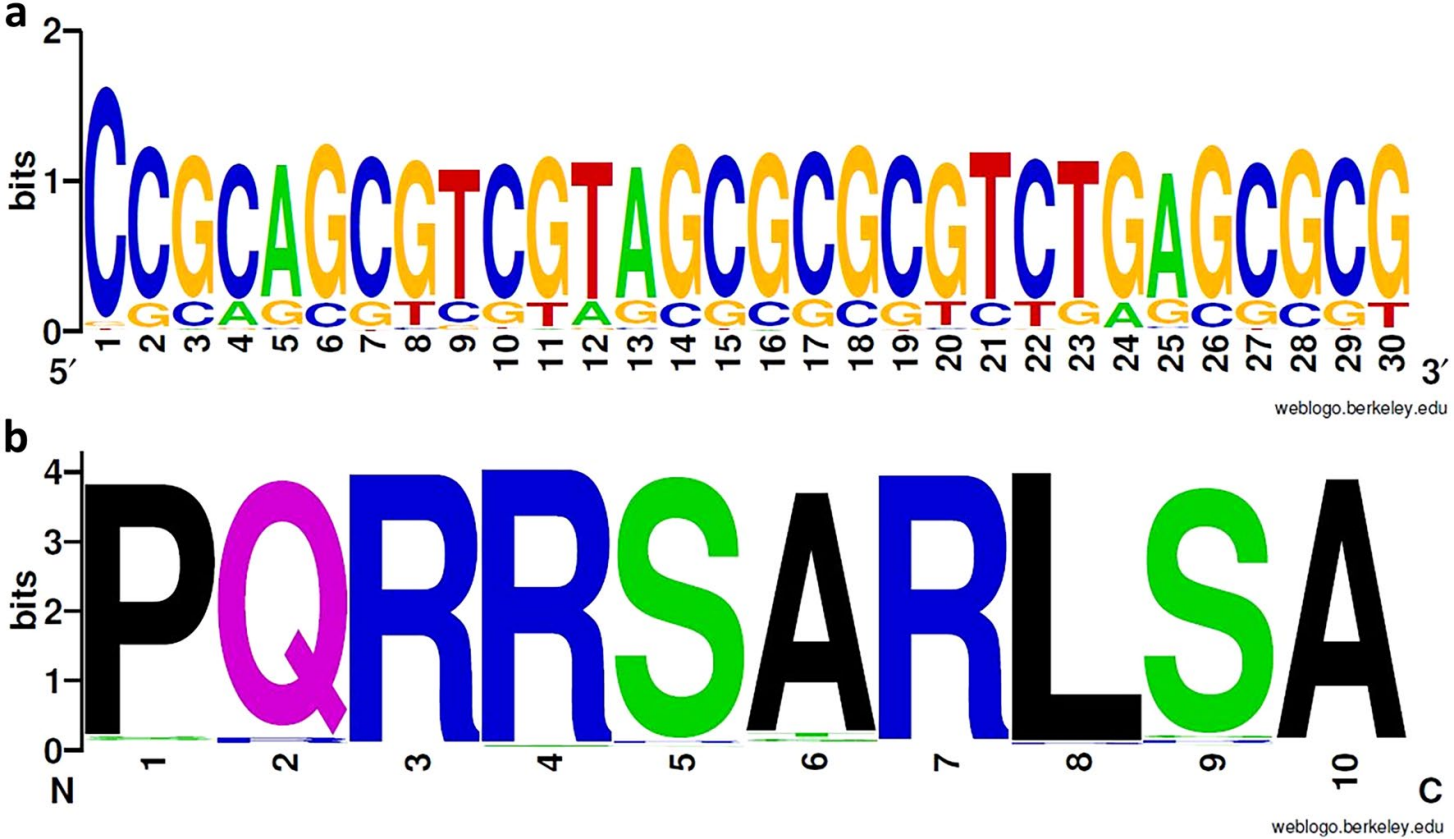
WebLogo representation of the merged CDR3 region nucleotide sequence (**a**) and amino acid sequence (**b**) from the post-selection pool. The height of each stack represents the conservation at that position, measured in bits, and the height of each symbol is proportional to its frequency at that particular position. C—Cysteine, G—Guanine, A—Adenine, T—Thymine, N—Unidentified nucleotide, P—Proline, Q—Glutamine, R—Arginine, S—Serine, A—Alanine, L—Leucine, X—Unidentified amino acid.

In the DNA library, reference CDR3 was found in 13,766 sequences, representing 43.95% of the merged reads. This corresponds to an increase in the percentage of sequences with the reference sequence, and an enrichment in these sequences. This was already expected, as the reference sequence continued to be the most frequent in the pre-selection antibody library (Fig. 1). The sequence fragment coding for the CDR3 has an average consensus of 81.48%, with PID values between 78.35 and 94.29% for each position, which reveals a higher consensus relative to the pre-selection library and, therefore, a decrease in diversity. Along the fragment, predominant nucleotides corresponded to the reference sequence (Fig. 7a).

In the translated peptide library, the reference CDR3 appeared in 6356 clusters, representing 76.15%. Positions 3, 4 and 8 demonstrated higher consensus (96.19%, 96.65% and 96.12%, respectively) than the remaining CDR3 (Fig. 7b), suggesting that these amino acids may be more involved in target binding, as they are prevalent in the selected pool. Nevertheless, the five most frequent mutations observed in the sorted library were transition from proline to serine in position 1 (2% of the clusters), alanine to tyrosine in position 6 (1.99% of the sequences), glutamine to arginine in position 2 (1.26%), arginine to glycine in position 4 (1.05%), and serine to cysteine in position 9 (0.99%).

For these five most frequent mutations, synonymous substitutions at the DNA level were analyzed, as the enrichment of different codons for the same amino acid residue after screening is indicative of a successful selection and validation of the present approach. Overall, there was an enrichment in all the codons for each selected amino acid, given by an increase in the frequency of their clusters from the pre-selection to the post-selection libraries (Table 2). Some codons were present in the pre- and/or post-selection libraries in frequencies below the sequencing error rate considered for MiSeq Illumina technology (< 0.1%)^52,60^. The enrichment of codons whose frequency in the post-selection library was below this threshold was not considered, as it may result from sequencing errors (Table 2). Notwithstanding, some codons had a clear enrichment after screening against nucleolin-overexpressing cells, such as the 65-fold increase in frequency of AGT coding for serine in position 1 of the CDR3, the fivefold increase in GGA for the glycine in position 4, and the 3.68-fold enrichment in ACC for tyrosine in position 6 (Table 2). On the other hand, three CDR3 amino acids remained highly conserved (PID > 95%): arginine in positions 4 and 7, and leucine in position 8. There was a clear representation of one codon for each of these positions, with a frequency higher than 85% in both pre- and post-selection libraries. Remaining codons were present in very low frequencies. There was no significant variation in codon frequency between the pre- and post-selection libraries for any triplet coding for the conserved positions, which is consistent with the corresponding high amino acid PID.

**Table 2:** Analysis of synonymous substitutions for the five most frequent mutations in the CDR3 region of the post-selection library.

Amino acid residue (Position in CDR3)	Corresponding DNA codon	Frequency in the pre-selection DNA library (%)^a^	Frequency in the post-selection DNA library (%)^b^	Enrichment (-fold)^c^
*Serine (1)*	TCT	0.01	0.04	4.00*
	TCC	0.05	0.12	2.40
	TCA	0.04	0.43	10.75
	TCG	0.33	2.21	6.70
	AGT	0.02	1.30	65.00
	AGC	1.25	1.60	1.28
*Tyrosine (6)*	ACT	0.00	0.03	0.03*
	ACC	0.11	0.40	3.64
	ACA	0.00	0.04	0.04*
	ACG	0.53	1.95	3.68
*Arginine (2)*	CGT	0.20	0.66	3.30
	CGC	0.25	0.72	2.88
	CGA	0.00	0.02	0.02*
	CGG	0.56	1.52	2.71
	AGA	0.00	0.04	0.04*
	AGG	0.23	0.46	2.00
*Glycine (4)*	GGT	0.18	0.20	1.11
	GGC	0.02	0.09	4.50*
	GGA	0.04	0.20	5.00
	GGG	0.04	0.08	2.00*
*Cysteine (9)*	TGT	0.02	0.04	2.00*
	TGC	0.36	0.86	2.39

^a^Percentage of clusters containing the specific codon at corresponding amino acid position within clustered nucleotide pre-selection library.

^b^Percentage of clusters containing the specific codon at corresponding amino acid position within clustered nucleotide post-selection library.

^c^Ratio between frequencies in the post-selection and pre-selection DNA libraries.

*Not significantly enriched.

### Selection and sequencing of individual clones

**Figure 8:**
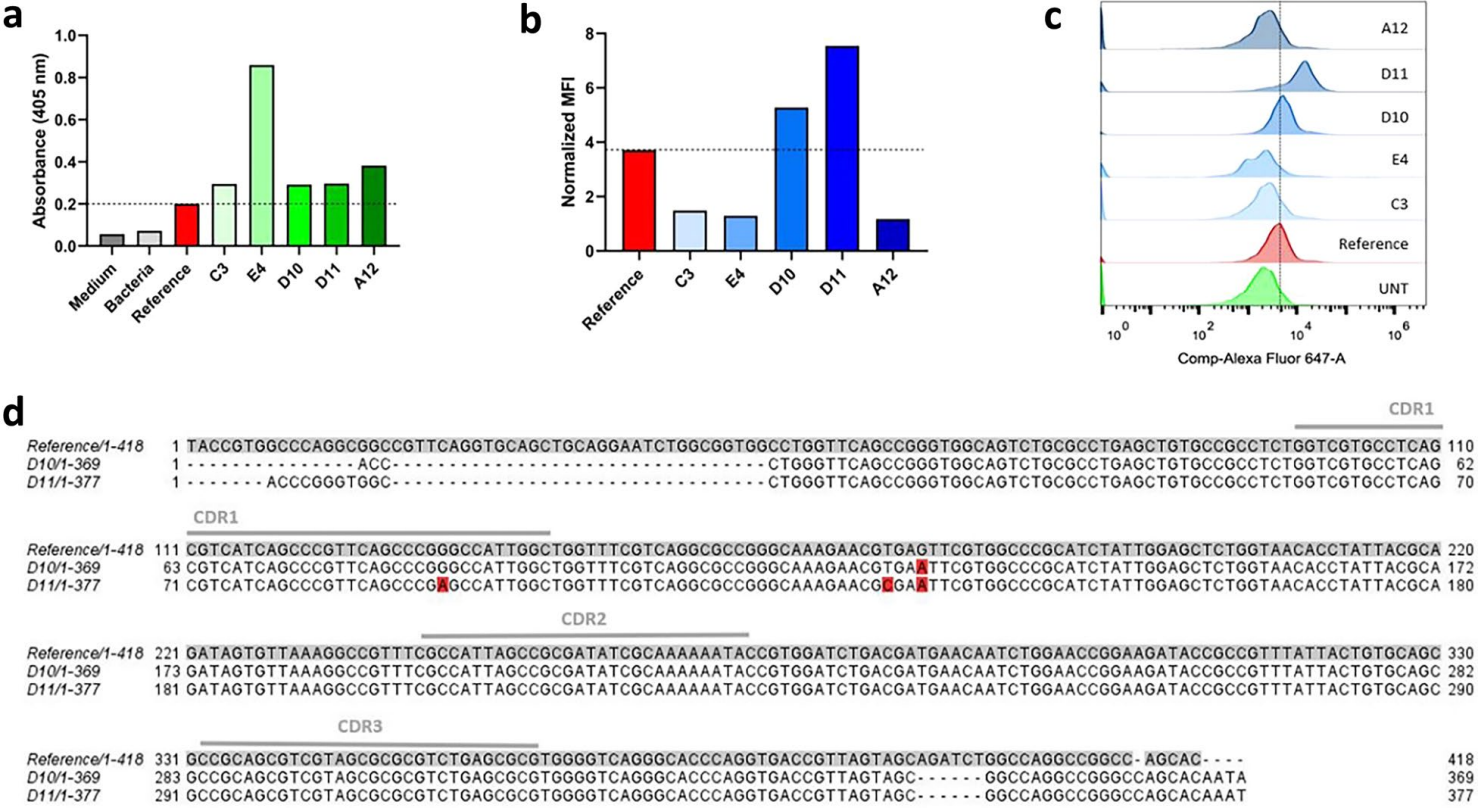
Selection and sequencing of VHH mutant antibodies. Five VHH mutant clones were selected by performing an expression and binding assay. Eighty-eight bacteria colonies from the plated post-sorter library were randomly picked, and used to express correspondent VHH mutant clones, by IPTG induction. Medium only, ER2738 bacteria without VHH coding plasmid, and bacteria bearing reference VHH coding fragment were used as controls. The five clones with the highest expression levels, given by increased values of Absorbance at 405 nm (**a**), were selected for a binding assay against MDA-MB-435S nucleolin-overexpressing cells. Flow cytometry assay was performed upon incubating the cells with 1 µg of each antibody for 45 min at 4 °C, followed by incubation with anti-HA-Alexa Fluor 647 secondary antibody, for 30 min at room temperature. Mean Fluorescence Intensity (MFI) normalized to cells incubated with secondary antibody only (**b**) and corresponding overlayed histograms of the green fluorescence signal (**c**) are presented. The two clones showing highest binding to nucleolin-overexpressing cells were sequenced and aligned against the reference VHH (**d**). Observed mutations are highlighted in red. Data from a representative experiment.

To further validate the phage-display screening by FACS, a preliminary assay of expression and binding was performed in parallel to the NGS analysis. Eighty-eight individual colonies were randomly picked from the plated post-sorter library, and used to produce the corresponding VHH clones by isopropyl-1-thio-β-D-galactopyranoside (IPTG) induction. Because colony choice was random, there was a chance that corresponding clones could be difficult to produce in a soluble format, impacting the analysis of their binding capacity^61^. Therefore, the five clones with higher levels of expression (Fig. 8a) from the induced microplate cultures were selected for a cytometry binding assay against nucleolin-overexpressing MDA-MB-435S cells. Two of these clones demonstrated increased binding to nucleolin-overexpressing cells relative to the reference antibody, specifically clone D10 with a 1.4-fold, and clone D11 with a 2-fold increase (Figs. 8b and c).

The two selected clones were then sequenced and aligned against the reference VHH. Clone D10 only presented one nucleotide mutation compared to the reference: a transition from guanine to adenine, in the Framework Region (FR) 2. Interestingly, clone D11 also shared that mutation. Additionally, it showed two other mutations: transition from guanine to adenine in the CDR1 region, and transition from thymine to cysteine in the FR2 (Fig. 8d). It must be highlighted that all three mutations are synonymous, as they code for the same amino acids as the corresponding nucleotide triplet in the reference sequence.

Furthermore, the frequencies of these sequences in the pre- and post-selection libraries were assessed. The sequence of clone D10 corresponded to 50.93% of the DNA clusters of the pre-selection library, which was enriched to 75.35% after the screening. Regarding clone D11, interestingly, its sequence represented 0.17% of the clusters in both libraries.

The fact that two randomly picked VHH clones were able to demonstrate higher binding to nucleolin-overexpressing cells relative to the reference antibody after only one round of panning, together with a clear enrichment in one of the selected sequences, supports the screening strategy developed herein.

## Discussion

In the last decades, monoclonal antibodies greatly improved the diagnosis and treatment of a variety of diseases. Their therapeutic effect is highly correlated with their specificity and affinity to a target, which explains the focus of antibody development on the optimization of their structure^62^. In this regard, antibody affinity can be synthetically modulated, by creating large and diverse antibody libraries, and screening them against the targets of interest, thus optimizing their therapeutic effect^14,26^.

An anti-nucleolin antibody was previously developed by our group^21^. With the ultimate goal of improving its biological activity, herein it is proposed the development of a fast and easy-to-perform affinity maturation strategy, combining a method of random mutagenesis that, to the best of our knowledge, has not been used for antibody library construction, and a cell-based phage display screening approach to select internalizing clones.

This in vivo random mutagenesis uses a bacteria strain harboring a low fidelity DNA polymerase I, and resulted in a library of 2.19 × 10^8^ transformants. Although antibody libraries can be achieved in orders of 10^10^ or higher in size^63^, the 10^8^ order of magnitude obtained with the mutagenesis used herein is acceptable for our objective. Firstly, as the starting point was an already functional antibody, we were mostly looking for a few mutations that could improve the antibody binding, without losing its inherent specificity to the target. Secondly, and despite a preferential selectivity of the polymerase for the first 750 bp from *ori*^22^, which includes the region where the reference antibody fragment was cloned (292–662 bp), this is an antibody fragment with a relatively small size (approximately 15 kDa, 350 pb), so there is a limited capacity for inducing mutagenesis in the antibody sequence. Lastly, in vivo mutagenesis is limited by bacterial transformation efficiency (6.93 × 10^8^ cfu/µg pUC19 for the electrocompetent JS200 bacteria used herein), and by the number of iterative rounds of mutagenesis performed, which can be increased if higher library sizes are desired.

Since a random mutagenesis method was used, it was important to assess the quality of the library, as the potential introduction of stop codons or shift mutations could impact the functionality of the clones. NGS has become a very useful tool for in depth characterization of antibody libraries^51^, particularly for antibody fragments such as the one used in here^50,64^. The fact that 78.29% of the sequences translated in frame 2 did not present stop codons supported the good quality of the library, in accordance with previous reports^52,65^. Additionally, only 4% of the DNA clusters presented a different length from the reference sequence, demonstrating that the majority of the mutations were nucleotide substitutions, in accordance with the mechanism of mutagenesis associated with low fidelity polymerase^22^.

Regarding library diversity, the 86% variability in DNA sequences, translated into 69% diversity in amino acid sequences (Table 1), likely reflected the redundancy of the genetic code, where each amino acid is codified by more than one nucleotide triplet (silent mutations). Despite this considerable percentage of variability, the most frequent sequence of the peptide library corresponded to the reference VHH. This was probably the result of mutations in regions of the plasmid outside the antibody fragment, or DNA sequences that resulted in the same reference peptide sequence, as mentioned before. Furthermore, the CDR3 reference sequence (i.e. the nucleolin targeting region), also appeared in approximately half of the library’s CDR3 regions. It is important to highlight that there is a considerable percentage of unidentified nucleotides in positions 3, 4 and 5 of the CDR3 sequence (Fig. 1a), resulting in 38.67% of unidentified amino acids in the second amino acid position, which may correspond to the reference sequence or different clones. Nonetheless, the diversity obtained is in accordance with the approximately 40–50% reported for other antibody fragment libraries^49,64^. This analysis demonstrated that the in vivo random mutagenesis was effective in the construction of an antibody library with an acceptable size, good quality, and functional variability.

The next step consisted in the screening of clones of interest by phage display against target cells. Phages displaying the reference antibody were used as control (Fig. 5a) for the selection of cells exhibiting the highest fluorescence signal (Fig. 5b). The small increase in fluorescence in one order of magnitude (Fig. 4b) observed for the population of cells incubated with library displaying phages relative to the cells incubated with reference phages (Figs. 4 and 5), is likely the result of a considerable presence of the reference CDR3 sequence in the library (at least 46.62%), as well the existence of mutations that may have not been beneficial for nucleolin-mediated binding and internalization. The increase in fluorescence suggested the existence of internalized phages displaying antibody clones that potentially had higher affinity to nucleolin. Despite this small shift observed, only one round of panning was performed. This decision resulted mainly from the reduced number of collected events, and consequent concern of a biased decrease in library diversity as a result of phage loss during the steps of cell lysis and bacteria infection needed for further rounds of selection. Additionally, it is known that the amplification of selected libraries can lead to a selective pressure and possibly culminate in the clonal dominance of antibodies with increased expression and display levels, but not necessarily high affinity to the target, not adding value to the enrichment process^64,66^. Moreover, given that post-selection NGS analysis demonstrated 25.56% of sequences frameshift mutations and/or stop-codons (Table 1), it was considered that phages displaying proteins resulting from these low-quality sequences would have growth advantages over the clones of interest. Additionally, by performing only one round of screening, the need for additional panning, reamplification, and phage labeling and titration steps is eliminated, and the selection method becomes much faster and simpler to execute, as aimed herein.

Finally, post-selection DNA was sequenced and compared to the initial library. At this point, increased diversity was no longer aimed, but rather a higher consensus in the sequences of the enriched pool. In this regard, the diversity of the post-selection pool decreased in about half (Table 1), and there was a decrease in the percentage of single occurrence sequences and an increase in sequences with more than one copy (Fig. 6). This suggested that many of the mutated antibodies with no binding and internalization advantage were eliminated upon only one round of selection, while there was an enrichment in potentially improved sequences. To our knowledge, the NGS analysis of Ravn et al. is the one that best correlates with ours from a methodological standpoint, as they compared the diversity of their original libraries with the pools obtained after each of three rounds of selection^65,67^. After only one panning, a decrease in single copy sequences between, approximately, 15% and 65%^65,67^, was reported. Regarding the quality of the post-selection pool, unexpectedly, the percentage of non-functional sequences slightly increased relative to the pre-selection library (25.56% vs 21.71%, respectively, Table 1), raising concerns of non-specific phage internalization. However, the use of several stringent conditions (FACS to select a narrow window of events with clear increased fluorescence signal, PBS supplemented with 2% FBS as blocking solution, washing between incubations at 4 °C and 37 °C, and acidic stripping of cell surface-bound phages, that were not internalized after incubation at 37 °C) decrease the potential of collection of phages internalized in a non-specific manner. Together with reports that only one round of panning may not be sufficient to decrease the percentage of non-functional sequences, such as sequences with stop codons^68^, it is suggested that such value is due to the quality of the constructed library, sequencing errors^69^, or bias during bacteria infection^49^.

Despite the enrichment, in accordance with the prevalence of the reference VHH sequence and CDR3 in the initial library and the aforementioned possibility of internalization by phage-displaying reference antibody, the reference CDR3 region still comprised 76.15% of the post-selection clusters. However, important information emerged from the CDR3 of the remaining antibodies collected. The sorted pool showed higher consensus in positions 3, 4 and 8 (arginine and leucine) (Fig. 7b), suggesting that these amino acids were likely more involved in target binding. In fact, it has been described that basic cationic amino acids, such as arginine, were involved in the binding and internalization of phages displaying a cell-penetrating antibody fragment^59^. Additionally, it has been demonstrated that F3 peptide is highly basic and is internalized by the NH_2_-terminal acidic domain of nucleolin^34^. Leucine is a non-polar amino acid, frequently present in VHH’s paratopes, stabilizing their hydrophobic core^70^. The five most frequent mutations observed in the sorted library indicated transitions to amino acids with more functional side chains, such as, proline and tyrosine (aromatic side chains), cysteine (sulfhydryl group), and arginine (long hydrophobic chain). Besides their potential relevance in the interaction with other functionalized amino acids in the nucleolin surface, these have been described as important in the stabilization of hydrophobic packed cores of other VHHs’ CDR3^71^. Differently, the transition from long side chain arginine to small amino acid glycine in position 6, can add some flexibility to the CDR3 loop, facilitating its binding to the target^72^. It is interesting to note that these represent mainly single mutations per sequence. In this regard, a single amino acid substitution from glutamine to lysine in the F3 peptide that composes the reference antibody CDR3 has led to a 90% lower binding extent to nucleolin-overexpressing cells^55^. This demonstration adds value to the potential of the selected pool, as it proves that even clones with minimal changes in their CDR3 can have significantly increased binding to the target.

Furthermore, the five most frequent CDR3 mutations were analyzed at the DNA level, showing an increase in frequency of certain codons for specific amino acid residues, indicating a selection towards specific CDR3 nucleotide sequences and corroborating the success of the screening protocol. Additionally, a preliminary expression and binding assay using randomly chosen clones was performed. It resulted in the selection of two mutant antibodies with higher binding to nucleolin-overexpressing cells relative to the reference VHH, confirming that the proposed method can lead to the selection of antibody hits. Sequencing of these clones demonstrated transition mutations relative to the reference antibody, in accordance with the mutation spectrum described for the JS200 mutator strain (80% of transitions, with a predominance of G to A mutations)^22^. However, the observed nucleotide transitions correspond to synonymous mutations, resulting in the same translated amino acids as the reference for the corresponding positions. Giving the latest suggestion that synonym mutations may not be silent, and impact proteins’ folding and structure^73,74^, we hypothesize that mutations in D10 and D11 resulted in antibodies with the same amino acid sequence but different folding and, therefore, binding performance relative to the reference^75^. The fact these mutations occurred in the CDR1 and FR2 regions corroborated the importance of the FR regions in VHH structure, thus assisting CDR3 binding to the target^70^. Furthermore, an enrichment in frequency of the clone D10 sequence from the pre- to the post-selection library was observed, adding to the validation of the screening strategy. Interestingly, clone D11 sequence presented the same low frequency (0.17%) before and after selection, despite showing the stronger binding to nucleolin-overexpressing cells. This corroborates previous reports that clone abundance and respective binding ability are not always correlated^76^.

As the discovery of new targets and strategies for antibody therapy demands for improvements in antibody development, the work described herein resulted in the validation of a novel antibody optimization strategy. It is a fast and simple approach for the creation and characterization of antibody libraries, and screening by phage display, that originated an antibody pool enriched with clones that potentially bind with higher affinity to nucleolin than the reference antibody, and are internalized by nucleolin-overexpressing cells, and that might be further used for affinity maturation in antibody development.

## Methods

### Reference anti-nucleolin VHH phagemid preparation

Reference anti-nucleolin VHH phagemid was obtained by insertion of the VHH coding fragment in a pComb3X vector, with restriction enzymes MfeI and FseI, and was provided by Synbio Technologies. Reference anti-nucleolin VHH coding fragment sequence was based on an anti-nucleolin antibody previously developed by our group^21^.

### In vivo random mutagenesis

An in vivo random mutagenesis method was used to create a VHH antibody library. Fifty µL of electrocompetent JS200 were transformed with 50 ng of reference anti-nucleolin-codifying plasmid in a 1 mm electroporation cuvette. Transformed bacteria were recovered in 1 mL Lysogeny Broth (LB) and grew 1 h at 37 °C and 220 rpm. Fifty µL of this culture were plated in LB-agar Petri dish containing 50 µg/mL chloramphenicol and 100 µg/mL ampicillin, and incubated overnight at 37 °C. Upon washing with 5 mL LB broth, colonies were then recovered, and DNA was extracted using ZR Plasmid Miniprep™-Classic (D4015, Zymo Research), following the manufacturer’s protocol. Isolated plasmid DNA was restricted with FastDigest HindIII restriction enzyme (FD0504, Thermo Fisher Scientific), to linearize the polymerase coding plasmid and ensure that only mutated VHH harboring plasmid was transformed in the next round of iteration. This restriction reaction was cleaned with Promega Wizard® SV PCR Clean-Up kit (A9281, Promega) for further transformation, and purified DNA was quantified in NanoDrop spectrophotometer. These steps were repeated in four iterative rounds.

### Construction of phage library

Phage library consists of phage particles displaying VHH antibody clones from the DNA mutated library. To create this library, five transformation reactions of 50 µL of electrocompetent ER2738 *E. coli* with 50 ng of purified DNA from the 4th round of mutagenesis were performed in parallel. After electroporation, each cuvette was flushed with 1 mL of Super Optimal Broth with Catabolite repression (SOC), and the combined 5 mL of transformed bacteria were incubated for 1 h at 37 °C and 250 rpm. Prewarmed (37 °C) Super Optimal Broth (SOB) (10 mL), with 100 µg/mL ampicillin and 10 µg/mL tetracycline, was added, and the resulting culture was incubated for two cycles of 1 h at 37°C and 250 rpm (where in the second, 4.5 µL of 100 µg/mL ampicillin were added).

For phage production, the culture was infected with 2 mL of M13KO7 helper phage (10^12^ pfu/mL), and the mixture was transferred to a 500 mL Erlenmeyer, together with 183 mL of prewarmed (37 °C) of SOB medium, including 100 µg/mL of ampicillin and 10 µg/mL of tetracycline. The resulting culture was incubated for 1.5–2 h at 37 °C and 300 rpm. Kanamycin (50 µg/mL) was then added, and the incubation further continued overnight at the referred conditions. The phage library was then recovered by precipitation: following centrifugation at 2400 g for 15 min at 4 °C, the supernatant was incubated with 4% (w/v) PEG_8000_/0.5 M NaCl, in ice, for 1 h, and centrifuged for 20 min at 4 °C and 8000 g; the phage pellet was washed with 3 mL solution of PBS, 15% Glycerol, and 1% BSA. The suspension was spun at 8000 g, for 5 min, at 4 °C and the supernatant was sterile filtered and stored at 4 °C. The same protocol was used to produce phages displaying reference anti-nucleolin VHH. Phages were titrated by counting plaque forming units (pfu) from dilution series of phage infected ER2738 bacteria.

### Phage labeling with pHrodo™ Green

Phages displaying both the mutated antibody library and the reference anti-nucleolin antibody were labeled with pH sensitive fluorogenic dye pHrodo™ iFL Green STP ester (P36012, Invitrogen). A phage solution (375 µL) was precipitated with 125 µL of a solution composed of 20% w/v PEG_8000_ and 2.5 M NaCl. The mixture was incubated in ice for 1 h and centrifuged for 10 min at 10,000 g. The resulting pellet was resuspended in 200 µL of 100 mM sodium bicarbonate, pH 8.5, and further incubated with 25 µL of 2 mM dye solution for 1 h at room temperature, protected from light. To purify the conjugated phages, the labeling reaction was precipitated twice with the previously solution, and resuspended in 400 µL of PBS. Labeled phages were stored at 4 °C, protected from light. Extent of phage labeling was assessed by coating a Nunclon Delta Surface black flat-bottom 96 well plate (165,305, ThermoFisher Scientific) with 10^8^ pfu of labeled reference-displaying phages/well, over 2 h, at 37 °C, protected from light. Then, wells were washed three times with PBS, and 50 µL of a pH 4 buffer solution (10,545,151, Fisher Chemical) was added to each well. pHrodo™ iFL Green STP ester has an excitation wave-length of 505 nm, and emission of 525 nm. Therefore, fluorescence was immediately read in a Varioskan LUX Multimode Microplate Reader (ThermoFisher Scientific). Controls included labeled phages incubated with PBS pH 7.4, and non-labeled phages incubated with either PBS pH 7.4 or pH 4 buffer solution.

### Preparation of DNA for Next Generation Sequencing

Plasmid DNA was isolated either from ER2738 bacteria (for library characterization) or phage (for selection analysis) using ZR Plasmid Miniprep™-Classic (D4015, Zymo Research), according to the manufacturer’s protocol. DNA concentrations and purity were assessed using a Nanodrop ND-1000 Spectrophotometer. Isolated DNA was used as template for amplification of the VHH fragments of the plasmid by PCR, using specific primers (FW 5′ TACCGTGGCCCAGGCGGCCCAGGTGCAGCTGCAGGAATCTG 3′; RV 5′ GTGCTGGCCGGCCTGGCCGCTACTAACGGTCACCTGGGTGC 3′). PCR reaction was performed with 500 ng of DNA, 0.5 µM of primers, 25 µL of NZYTaqII 2 × Green Master Mix (MB358, NZYTech), and nuclease-free water, up to a final volume of 50 µL. Thermocycler (Thermoblock VWR 732-1210-Doppio) was programmed with one initial denaturation step of 1 cycle of 3 min at 95 °C, followed by 35 cycles of denaturation (30 s at 94 °C), annealing (30 s at 56 °C), and extension (30 s at 72 °C). One cycle of final extension (10 min at 72 °C) was performed and held at 4 °C. VHH fragments resulting from PCR were purified using the QIAquick Gel extraction kit (28,704, QIAGEN) by adapting the protocol for PCR cleaning according to manufacturer’s instructions. Cleaned PCR product was quantified using a Nanodrop ND-1000 Spectrophotometer.

### Next Generation Sequencing (NGS)

Next Generation Sequencing (NGS) and sequence data processing were performed at Genoinseq (Cantanhede, Portugal). For Illumina sequencing, indexes and adaptors were added to the samples by PCR amplification. The PCR product was purified and normalized using SequalPrep 96-well plate kit (ThermoFisher Scientific) and paired-end sequenced in the Illumina MiSeq^®^ sequencer with the V3 chemistry, according to manufacturer’s instructions (Illumina). The obtained raw reads were extracted from Illumina MiSeq^®^ System in fastq format and quality-filtered with PRINSEQ version 0.20.4 to remove sequencing adapters, reads with less than 150 bases and trim bases with an average quality lower than Q25 in a window of 5 bases. Then, the forward and reverse reads were merged by overlapping paired-end reads with AdapterRemoval version 2.1.5 using default parameters. Frame 2 of the nucleotide sequences were translated into amino acids using EMBOSS Transeq version 5.0.0. Sequences containing stop codons were filtered out. Peptide sequences were then clustered at an identity of 100% with CD-HIT version 4.6.1. The cluster representative sequences were aligned using MAFTT version 7.221.3. The alignments included the reference sequence provided. Nucleotide sequences were also clustered in CD-Hit at 100% similarity to provide sequence diversity at the nucleotide level. Cluster nucleotide representative sequences were aligned with MAFFT and included the reference sequence provided. Jalview software was used for visualization and analysis of library alignment.

### Cell culture

MDA-MB-435S (ATCC^®^ HTB-129TM) cell line was cultured in RPMI 1640 with L-glutamine (VWR Life Science) supplemented with 10% (v/v) heat-inactivated Foetal Bovine Serum (FBS) (Biowest, France), and 1% (v/v) Antibiotic Antimycotic Solution 100X (10,000 units penicillin, 10 mg streptomycin and 25 μg amphotericin B per mL) (Corning). Cells were maintained at 37 °C in a humidified atmosphere of 5% CO_2_.

### Internalizing cell-based phage display

Two hundred and fifty thousand MDA-MB-435S nucleolin-overexpressing cells were first washed with a solution of PBS and 2% FBS to decrease non-specific binding, followed by incubation with 10^8^ pfu of pHrodo-labeled phages, displaying either reference anti-nucleolin VHH or the mutated antibody library, for 45 min at 4 °C, to promote antibody binding to the target. Upon washing twice with PBS to remove non-specific binders or binders with low affinity, cells were then incubated for 1.5 h at 37 °C, a condition that favors nucleolin-mediated internalization. Surface-bound phages were removed with 100 mM glycine, 150 mM NaCl, pH 2.5 for 5 min at room temperature, followed by washing with neutralizing buffer (Tris–HCl 1 M pH 7.4). After a washing step with PBS, cells were sorted in a BD FACSAria III flow cytometer. Cells associated with events with a fluorescence signal higher than the ones from cells incubated with reference antibody-displaying phages, were collected in PBS. These were then incubated with NP-40 cell lysis buffer (150 mM NaCl, 1% NP-40, 50 mM Tris–HCl, pH 7.4) for 15 min in ice and vortexed every 5 min in between. The mixture was centrifuged at 16 000 g for 10 min at 4 °C and the lysate was used to infect 200 µL of ER2738 bacteria, for 15 min at room temperature. The resulting mixture was plated in LB-agar Petri dish containing 100 mg/mL ampicillin, in a total of four plates, for further DNA isolation and library analysis.

### Selection and sequencing of individual clones

An expression and binding assay was performed to select five random mutant clones. Accordingly, eighty-eight colonies of ER2738 *E. coli* were randomly picked from the plated post-sorter library, and cultured in a 96-well plate (3788, Corning^®^) with 200 µL of Super Broth (SB) medium, overnight, at 37 °C and 180 rpm. ER2738 bacteria without VHH coding plasmid, and bacteria bearing reference VHH coding fragment were used as controls. On the following day, culture was diluted 1:100 in SB medium with ampicillin (100 mg/L) and grown at 37 °C, 180 rpm, until reaching an OD between 0.7 and 0.9, at 600 nm. Expression was induced with 1 mM IPTG (Thermo Scientific), for 16 h, at 30 °C and 180 rpm. Plate was then centrifuged, and the resulting pellet was resuspended in a solution of BugBuster^®^ MasterMix (Merck Millipore) and cOmplete™ Protease Inhibitor Cocktail (Roche), followed by incubationat room temperature for 20 min. Then the plate was centrifuged, and the resulting supernatant, containing the antibody fragments, was added to a high binding surface 96 well plate (3690, Corning^®^). After incubation for 1 h at 37 °C for adsorption to the plate, wells were washed with PBS and blocked with 100 µL of PBS with 3% BSA, for 1 h at 37 °C. VHH expression was detected with anti-HA-peroxidase antibody (Roche), using ABTS substrate (Merk Millipore). Absorbance at 405 nm was measured on Model 680 microplate reader (Bio-Rad). The five clones with highest absorbance, as well as the reference VHH, were then produced in a 10 mL culture volume, using the same IPTG-induction protocol as before. Antibodies were purified using Dynabeads™ His-Tag Isolation & Pulldown (Invitrogen), according to the manufacturer’s protocol, and Bradford assay was used to quantify the amount of protein produced, using Bovine serum albumin (BSA) as a standard. Binding was assessed by flow cytometry against nucleolin-overexpressing MDA-MB-435S cells. Briefly, 1.5 × 10^5^ cells were washed twice with ice cold PBS with 1% BSA, and incubated with 1 µg of each antibody, for 45 min at 4 °C. Cells were then washed twice with ice cold PBS with 1% BSA, and incubated with anti-HA-Alexa Fluor 647 secondary antibody (sc-7392 AF647, Santa Cruz), in a 1:500 dilution, for 30 min at room temperature. After two more washes with ice cold PBS with 1% BSA, cells were resuspended with PBS, and 20 000 events per sample were acquired on Cytek^®^ Aurora flow cytometer. The two clones with higher binding to nucleolin-overexpressing cells were sequenced by Sanger Sequencing technology using the Mix2Seq Service provided by Eurofins Genomics (Ebersberg, Germany). Briefly, samples were prepared following the “Preparation of DNA for Next Generation Sequencing” protocol and then submitted. Resulting sequences were aligned against the provided Reference VHH sequence, and Jalview software was used for visualization and analysis.

## Data Availability

The sequencing datasets generated during and/or analyzed during the current study relate with synthetic libraries constructed by us, currently being used in further research with the purpose of developing therapeutic proteins. As such, the raw required to reproduce these findings cannot be shared at this time as the data also forms part of an ongoing study.
